# Involvement of the Macrophage Migration Inhibitory Factor (MIF) in Lipedema

**DOI:** 10.3390/metabo13101105

**Published:** 2023-10-23

**Authors:** Mauro Vasella, Stefan Wolf, Eamon C. Francis, Gerrit Grieb, Pablo Pfister, Gregory Reid, Jürgen Bernhagen, Nicole Lindenblatt, Epameinondas Gousopoulos, Bong-Sung Kim

**Affiliations:** 1 Department of Plastic Surgery and Hand Surgery, University Hospital Zurich, 8091 Zurich, Switzerland; 2 Department of Plastic and Reconstructive Surgery, Guys and St Thomas Trust, London SE1 7EH, UK; 3 Department of Plastic Surgery and Hand Surgery, Gemeinschaftskrankenhaus Havelhoehe, 14089 Berlin, Germany; 4 Department of Plastic Surgery, Hand Surgery and Burn Center, University Hospital RWTH Aachen, 52074 Aachen, Germany; 5 Department of Surgery, Stadtspital Zürich Triemli, 8063 Zurich, Switzerland; 6 Division of Vascular Biology, Institute for Stroke and Dementia Research (ISD), Ludwig-Maximilians-University (LMU), 81377 Munich, Germany; 7 Munich Cluster for Systems Neurology (SyNergy), 81377 Munich, Germany; 8 Munich Heart Alliance, German Centre for Cardiovascular Diseases, 80802 Munich, Germany

**Keywords:** lipedema, macrophage migration inhibitory factor, CD74, cytokine

## Abstract

Lipedema is a chronic disorder that mainly affects women. It is often misdiagnosed, and its etiology remains unknown. Recent research indicates an accumulation of macrophages and a shift in macrophage polarization in lipedema. One known protein superfamily that contributes to macrophage accumulation and polarization is the macrophage migration inhibitory factor (MIF) family. MIF-1 and MIF-2 are ubiquitously expressed and also regulate inflammatory processes in adipose tissue. In this study, the expression of MIF-1, MIF-2 and CD74—a common receptor for both cytokines—was analyzed in tissue samples of 11 lipedema and 11 BMI-matched, age-matched and anatomically matched control patients using qPCR and immunohistochemistry (IHC). The mRNA expression of MIF-1 (mean 1.256; SD 0.303; *p* = 0.0485) and CD74 (mean 1.514; SD 0.397; *p* = 0.0097) were significantly elevated in lipedema patients, while MIF-2 expression was unaffected (mean 1.004; SD 0.358; *p* = 0.9718). The IHC analysis corroborated the results for CD74 expression on a cellular level. In conclusion, our results provide first evidence for a potential involvement of the MIF family, presumably via the MIF-1-CD74 axis, in lipedema.

## 1. Introduction

Lipedema is defined as a chronic disorder that mainly affects women, with an estimated prevalence in the female population at around 10% [1,2,3]. Lipedema is classified according to stages I–III and IV when accounting for a mixed lipo-lymphedema type and according to morphology (I–V) [1,4]. First onset of clinical manifestations and symptoms is usually linked with stages of hormonal changes such as puberty, pregnancy or menopause [1]. Typically, patients will present a disproportionate distribution of adipose tissue in a bilateral symmetrical manner of the extremities without affecting the trunk, hands or feet [2]. Other clinical features include pain, tenderness, tendency of bruise, hematoma formation and, most importantly, resistance to mass reduction through exercise or dietary measures [4]. Unfortunately, it still remains a relatively under- or misdiagnosed condition, sometimes being mistaken for obesity.

While the exact etiology remains unknown, many factors have been suggested to contribute to the development of lipedema. One reported factor is genetic predisposition, with over 50% of the affected patients having at least one first-degree relative suffering from lipedema [1,5]. Other possible contributors have been identified by Al-Ghadban et al. in a study comparing adipose and skin tissue from patients with and without lipedema [6]. The authors observed a significant increase in adipocyte area, number of macrophages in adipose and skin tissue, number of blood capillaries and increased capillary diameter in lipedema patients. However, neither lymphangiogenesis nor increased numbers of T cells were observed. It was hypothesized that lipedema patients suffer from a capillary structural defect with the excess fluid provoking a hypertrophy of the adipocytes, causing an inflammation. In return, the inflammation provokes nerve fibers and fibrosis, resulting in pain and the impaired possibility of weight loss. We not only confirmed these findings recently in ten lipedema patients but also reported an aberrant lipid metabolism as well as gene expression that were associated with macrophages [7]. Regarding the involved molecular players in lipedema, Siems et al. found a marked increase in vascular endothelial growth factor (VEGF)-A, which is a known inducer of angiogenesis [8]. More recently, we discovered increased levels of VEGF-C, a potent inducer of lymphangiogenesis, and a decrease in VEGF-A and VEGF-D levels in lipedema patients [9]. By analyzing gene expression, we identified a downregulation of Tie2 and FLT4 (VEGFR-3), which is coherent with increased macrophage infiltration. However, we interestingly did not observe morphological changes in lymphatic vasculature. These findings suggest that macrophage-derived cytokines known to be involved in inflammatory processes, angiogenesis, lymphangiogenesis and other functions such as metabolism and the recruitment of immune cells might play an important role in the development of lipedema.

Another distinct characteristic of lipedema is the increased population of M2-polarized macrophages and low-grade inflammation [9]. The generally accepted nomenclature for M1 and M2 polarization is “classically activated” and “alternatively activated”, respectively. M1 macrophages are considered pro-inflammatory, whereas M2 macrophages take on a more anti-inflammatory role [10]. Interestingly, this aspect is distinct from the classic inflammatory state of adipose tissue in obese patients, which is mostly composed of M1 polarized macrophages [10]. However, lipedema and obese patients share common traits like adipose tissue hypertrophy and the accumulation of inflammatory cells, especially macrophages [11].

In this context, the MIF superfamily, which is composed of cytokines and receptors playing crucial roles in the recruitment of macrophages and their polarization, might be of significant importance in lipedema [12,13,14].

The macrophage migration inhibitory factor (MIF-1), which was first described over half a decade ago, is a pleiotropic cytokine involved in many immune and inflammatory processes [15]. MIF-1 is widely expressed and is a known upstream mediator of innate immunity and inflammatory regulator in the human body. MIF-1 has the ability to mediate its functions by interacting with cluster of differentiation 74 and 44 (CD74/CD44) as well as the chemokine (CXC)-motif receptors CXCR2, CXCR4 and CXCR7 or receptor-independently through JAB1 [16,17,18,19,20].

Although described as early as 1995 [21], the true function of the D-dopachrome tautomerase (D-DT or MIF-2) was only recently reported. It acts as a structural and functional homolog to MIF-1 [22]. MIF-2 shares pro-inflammatory features with MIF-1 in diseases and conditions such as sepsis and also binds to CD74 [23].

Despite its homologous action, other studies have revealed distinct or even inverse MIF-1/-2 action in cardiovascular disease, autoimmune disease, obesity, adipose tissue inflammation and wound healing [11,13,22,24,25]. More specifically, MIF-1 promotes while MIF-2 alleviates adipose tissue inflammation [11].

Given the predominant presence of macrophages in lipedema, an involvement of the MIF axis regarding the pathophysiology of lipedema seems logical. In this study, we evaluated the expression of MIF-1, MIF-2 and their receptors CD74, CXCR2 and CXCR4, which were investigated in lipedema patient samples, suggesting their possible involvement in the development of this still not fully understood and underdiagnosed disease.

## 2. Materials and Methods

### 2.1. Patients

Our study was conducted according to the principles of the Declaration of Helsinki, and the protocols have been approved by the Ethical Committee of the University Hospital Goettingen, Goettingen, State of Lower Saxony, Germany (Nr. 23-11-17), prior to patient recruitment. Prior to surgery, patients had been informed in detail in oral and written form and had provided their written informed consent. Tissue samples were collected from lipedema and control female patients, which were BMI-matched, age-matched and anatomically matched by harvesting them from the proximal portion of the thighs. Lipedema was diagnosed according the criteria of Wold et al., which was also used in our previous study [26,27]. These criteria were met by all patients included in this study.

### 2.2. Tissue Collection

The collection of tissue samples was conducted during the surgical procedure, which was performed via liposuction. The samples were directly fixed for a duration of 4 h in 4% PFA/PBS at 4 °C. Afterwards, the specimens were embedded in paraffin and were cut for a histopathological analysis.

### 2.3. RNA Extraction and qPCR

RNA isolation and cDNA transcription was performed according to the same protocol used in one of our earlier studies [9]. The primers used for the qPCR analysis are provided in Table S1. The applied protocol and thermal cycler were the same as in the aforementioned study, which included the use of the RNeasy Lipid Tissue Mini Kit (Qiagen, Hilden, Germany) for RNA isolation, cDNA RT2 First Strand Kit (Qiagen, Hilden, Germany) for cDNA transcription, FastStart SYBR Green Master Mix (Roche, Basel, Switzerland) for the PCR reactions and CFX 96 C100 Thermal Cycler (Bio-Rad Laboratories, Hercules, CA, USA) [9]. GAPDH and B2M served as housekeeping genes, and the calculation of the fold changes of gene expression was performed using the ΔΔCT method [28]. The qPCR results of all target genes were normalized against each of the two housekeeping genes.

### 2.4. Immunohistochemistry and Histology

Staining was performed using an automated IHC system on formalin-fixed and paraffin-embedded tissue of representative sections that were cut into 2 μm thick samples according to the protocol used in a study of the Institute of Pathology and Molecular Pathology, University Hospital Zurich, using a Ventana Benchmark XT automated staining system (Roche Tissue Diagnostics, Basel, Switzerland) [29]. The samples were loaded either in the Bond-Max system (Leica Microsystems, Wetzlar, Germany) or BenchMark Ultra system (Roche Tissue Diagnostics, Basel, Switzerland).

The following antigenes were targeted with antibodies: MIF-1 (Abcam, Cambridge, UK), CXCR2 (Thermo Fisher, Waltham, MA, USA) and CXCR4 (Abcam, Cambridge, UK). MIF-2 and CD74 antibodies were provided by the Bernhagen lab, 81377 Munich, Germany.

The staining assessment was performed according to the study of He et al. [30]; it included the Remmele score, which is calculated by multiplying the intensity and percentage of staining. Expression was considered negative or low when the score ranged from 0 to 2 and positive or strong when the score was >2.

### 2.5. Statistical Analysis

The statistical analysis was performed as in one of our previous publications [27] using GraphPad Prism V8.0 (GraphPad Software, San Diego, CA, USA). A linear regression analysis was performed using the Pearson correlation coefficient and two-tailed *p*-values. A non-parametric unpaired Mann–Whitney U test was performed for non-Gaussian distribution, whereas a two-tailed Student’s *t*-test was performed for Gaussian distribution. Sample sizes and statistical analyses are indicated in the figure legends unless otherwise mentioned. *p* < 0.05 was accepted as statistically significant when compared with BMI-matched, age-matched and anatomically matched control patients.

## 3. Results

### 3.1. Patients

A total of 22 patients were selected for tissue analysis, all of which were female. The patients included 11 diagnosed with lipedema (L) and 11 control (C) patients. Both cohorts were age-matched, BMI-matched and anatomically matched. The patient characteristics are summarized in Table 1.

### 3.2. Expression Analysis Using qPCR and Correlation with BMI

**Figure 1:**
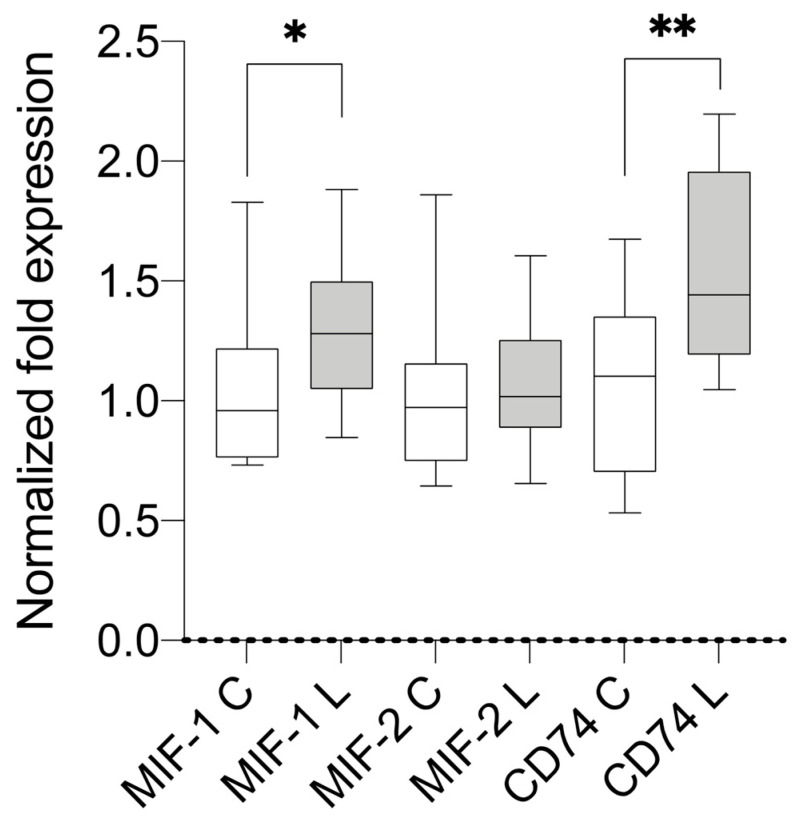
Results of mRNA expression levels in the subcutaneous tissue of lipedema (L; n = 11) and control patients (C; n = 11). * signifies *p* ≤ 0.05 and ** *p* ≤ 0.01.

**Figure 2:**
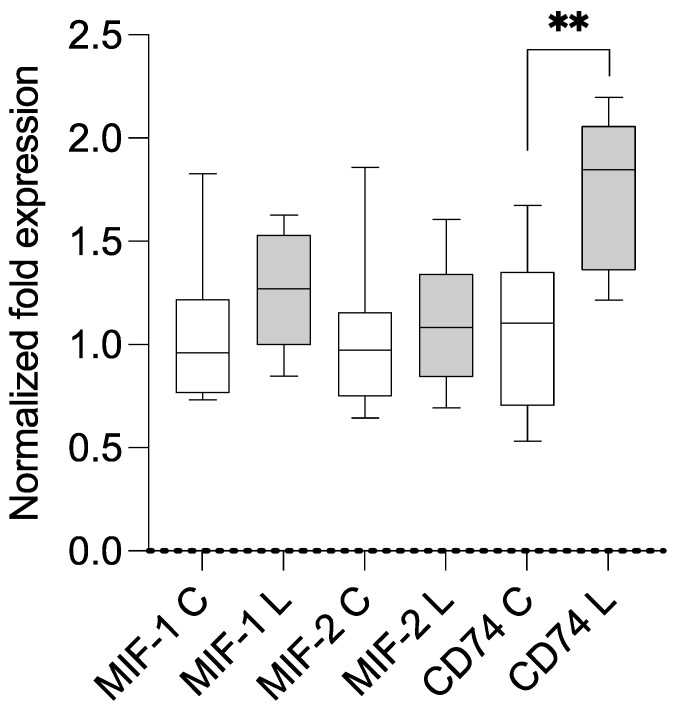
Results of mRNA expression levels in the subcutaneous tissue of stage II lipedema (L; n = 5) and control patients (C; n = 11). ** signifies *p* ≤ 0.01.

**Figure 3:**
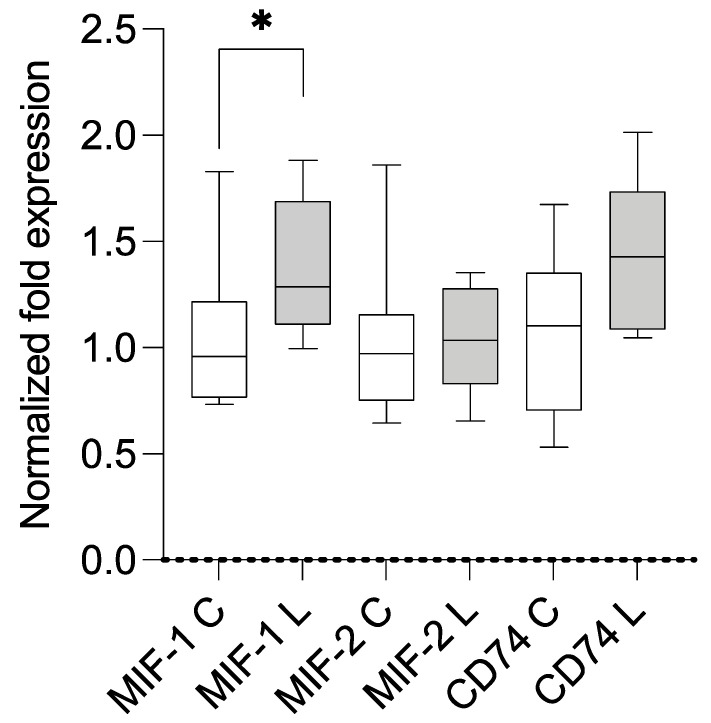
Results of mRNA expression levels in the subcutaneous tissue of stage III lipedema (L; n = 6) and control patients (C; n = 11). * signifies *p* ≤ 0.05.

The samples of both groups were analyzed using qPCR (primers are summarized in Table S1). Compared with the housekeeping genes and control group, the lipedema patients showed a statistically significant overexpression of *MIF-1* (mean 1.256; SD 0.303; *p* = 0.0485) and *CD74* (mean 1.514; SD 0.397; *p* = 0.0097). A separate comparison of stage II and III lipedema with the control group resulted in a significant overexpression of *CD74* in stage II lipedema (mean 1.703; SD 0.3959; *p* = 0.0071) and a significant overexpression of *MIF-1* in stage III lipedema (mean 1.370; SD 0.3266; *p* = 0.0383). The results are illustrated in Figure 1, Figure 2 and Figure 3. A linear regression analysis was performed, and the Pearson correlation coefficient (r) and *p*-values (two-tailed) were determined to establish if there was a correlation between BMI and MIF-1 as well as CD74; there were no significant correlations. MIF-1 showed r-squared values of 0.06777 for the control group and 0.003002 for the lipedema group. The *p*-values were 0.4987 (C) and 0.8805 (L). For CD74, the r-squared values were 0.09642 (C) and 0.05269 (L), and the *p*-values were 0.4161 (C) and 0.5524 (L). The results are illustrated in the Supplementary Materials (Figure S1).

### 3.3. Immunohistochemistry (IHC)

**Figure 4:**
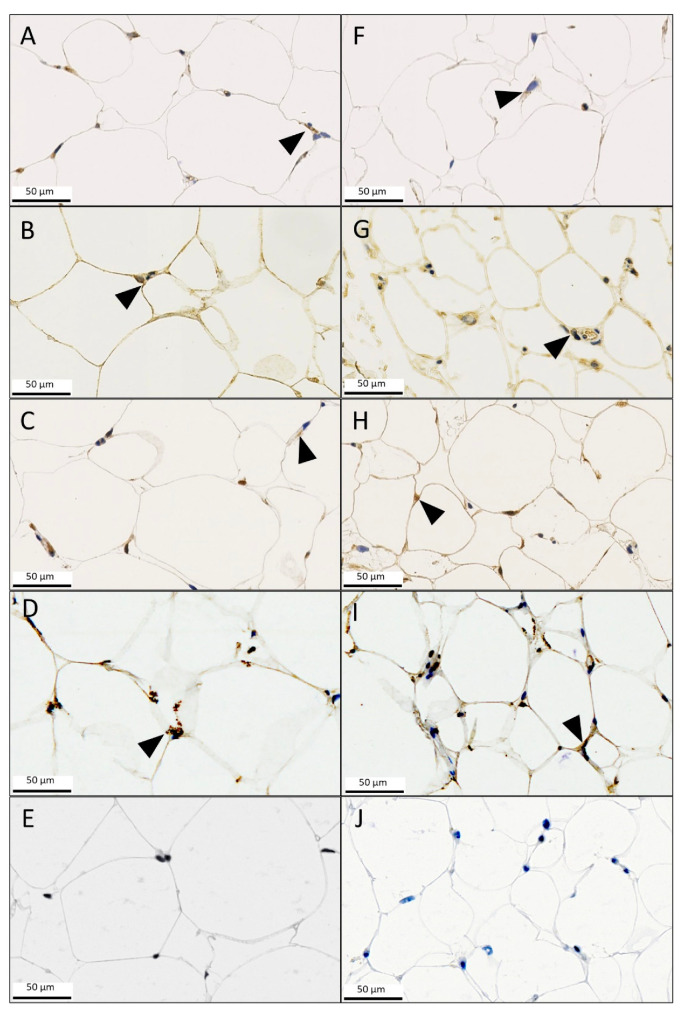
Immunohistochemistry subcutaneous adipose tissue of a lipedema case (**A**–**E**) and control patient (**F**–**J**). Arrowheads always indicate the expression of the stained protein. (**A**,**F**) Staining of CD74. (**B**,**G**) Staining of the widely expressed MIF-1 in the cytoplasm of adipocytes and macrophages as well as in secreted form. (**C**,**H**) Staining of MIF-2 and (**D**,**I**) CXCR2, also in the cytoplasm of adipocytes and macrophages as well as in secreted form, with the latter showing strong expression for both groups. (**E**,**J**) Very weak staining of CXCR4 in both groups.

**Figure 5:**
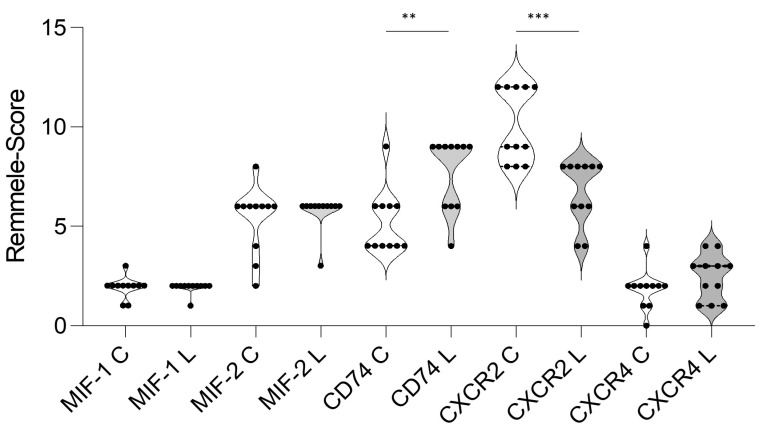
Results of IHC scores of CD74, MIF-1, MIF-2, CXCR2 and CXCR4 staining in the subcutaneous tissue of lipedema (L; n = 11) and control (C; n = 11) patients. ** signifies *p* ≤ 0.01 and *** *p* ≤ 0.001.

A histopathological analysis was performed to further investigate the increased MIF-1 and CD74 levels of the lipedema tissue samples. MIF-1, MIF-2, CD74, CXCR2 and CXCR4 staining were employed (Figure 4). The staining showed no significant difference in scores for MIF-1 (mean (L) 1.909 ± 0.302 and (C) 1.909 ± 0.539; *p* = 0.99), MIF-2 (mean (L) 5.727 ± 0.905 and (C) 5.363 ± 1.690; *p* = 0.5384) and CXCR4 (mean (L) 2.455 ± 1.128 and (C) 1.818 ± 0.982; *p* = 0.2137) expression in the cytoplasm and membrane of adipocytes and in the macrophages between the two groups, as illustrated in Figure 4. However, the histological analysis of CD74 did confirm the results of overexpression in lipedema patients (mean (L) 7.727 ± 1.849 and (C) 5.181 ± 1.601; *p* = 0.0026). Furthermore, expression of CXCR2 was significantly increased in the control group (mean (L) 6.727 ± 1.618 and (C) 10.09 ± 1.868; *p* = 0.0002). The results and IHC expression scores are summarized in Figure 5.

## 4. Discussion

We were able to highlight a statistically significant overexpression of MIF-1 and its receptor CD74 on an mRNA level in the subcutaneous adipose tissue of lipedema patients. This is in line with the findings of previous studies that emphasized an increase of MIF expression in inflamed adipose tissue [10,14,31]. Furthermore, we were also able to confirm that MIF-2 was unaltered, which interestingly is not completely congruent with the current literature regarding adipose tissue inflammation in obesity, wound healing and autoimmune disorders [13,32,33]. The precise function of MIF-1 and CD74 in lipedema remains unclear. MIF-1 is known to be secreted by preadipocytes, mature adipocytes and macrophages of the adipose tissue and promotes size and number increase in mature adipocytes as well adipogenesis [11]. The MIF superfamily is a known key driver in the polarization and recruitment of macrophages [12], which led us to theorize that it could be involved in the development or, at least, sustainment of lipedema, therefore making it of significant relevance. It is ubiquitously expressed and functions as an upstream mediator of inflammatory processes [15]. The members include MIF-1, MIF-2 (D-DT) and their main receptor CD74. Both MIF-1 and MIF-2 are produced by macrophages and stored in preformed vesicles for rapid release in the presence of inflammatory stimuli, and they have an autocrine effect on macrophages. Our IHC stainings did confirm an increase in CD74 expression, whereas MIF protein expression appeared unaltered when compared with the control group. This discrepancy may be rooted in the constitutive MIF storage in preformed intracellular pools, which is rapidly released upon stimulation and therefore not reliably measured in samples [34,35]. Earlier studies revealed increased protein and mRNA levels of MIF-1 in inflamed subcutaneous adipose tissue. Moreover, the MIF superfamily plays an important role in the cell mobilization and polarization of macrophages during inflammation [31,36]. In fact, MIF-1 is a known M1-polarizing pro-angiogenic factor [11,36]. It is known to recruit inflammatory macrophages; moreover, overexpression is associated with a worse outcome in sepsis [12]. As already mentioned above, a deficiency in MIF-2 has a pro-inflammatory effect; however, it rather alleviates inflammation in adipose tissue and does not possess the same effect on the migration of macrophages like MIF-1, which is achieved through the activation of CXCR2, amongst others [11,12,13].

However, when considering unchanged MIF-2 levels, an MIF-1-driven CD74 activation contributing to adipose tissue inflammation and macrophages skewed towards the M1 phenotype may be postulated. This hypothesis, of course, demands further elucidation. Chan et al. recently reported M1 polarization by CD74 and related insulin resistance and adipose tissue inflammation [37]. However, the adipose tissue inflammation cascade through MIF-CD74 may be context-dependent as our earlier studies have provided evidence for a downregulation of CD74 in inflamed adipose tissue from critical wounds [24]. How MIF-1 and CD74 promote the adipose tissue hypertrophy in lipedema is also unknown. However, it was shown that CD74, which builds a complex with CD44, fosters cell survival and inflammation [23,38], which may apply to adipose tissue in lipedema patients. The staining of CXCR4 demonstrated low expression rates for both groups. Yao et al. showed that a deficiency in CXCR4 actually exacerbated obesity and exacerbated chronic adipose inflammation via increased macrophage infiltration, and it even compromised the thermoregulation of brown adipose tissue in a mouse model [39]. Our selected population is obese by definition, which could explain the low or very low expression of CXCR4. On the other hand, CXCR2 showed strong positive expression for both groups; however, it was significantly increased in the control group. CXCR2 expression on blood neutrophils was found to be significantly increased in obese patients in a study conducted by Kopasov et al. in which they took samples from the subcutaneous tissue and blood of patients undergoing an abdominoplasty [40]. They hypothesized that the increased expression of chemokines and chemokine receptors in obese patients could be a possible predisposition for postoperative complications like impaired wound healing due to the chronic inflammation already present in the subcutaneous adipose tissue. Further in vivo studies discovered using mouse models that inducting CXCR2 was found to worsen steatohepatitis [41] and antagonizing CXCR1 and CXCR2 protected mice from metabolic disease by a modulation of the inflammatory process [42]. Moreover, Dyer et al. were able to determine that knocking out CXCR2 in mice resulted in a significantly reduced number and size of subcutaneous adipocytes, further underlining the role of the MIF superfamily in adipogenesis [43].

Lipedema is a chronic disorder with a typically disproportionate distribution of adipose tissue in the lower extremities, mainly affecting women [1,2,3]. Diagnosis remains difficult to this day as it is frequently confounded with obesity; however, we have been able to demonstrate significantly increased serum levels of cholesterol, low-density lipoprotein and triglycerides in a previously conducted study [7]. The specific etiology and pathophysiology of lipedema remains unknown. An increasing number of publications suggest the critical presence and potential role of macrophages in lipedema. This is depicted through the histologic assessment and expression profile analysis of subcutaneous tissue and through the quantitative evaluation of systemically circulating cytokines [6,7,27]. In terms of cytokines, it has been previously shown that lipedema patients have increased levels of interleukin (IL) 11, IL-28A and IL-29, with the latter two being known to be secreted by macrophages; however, IL-6, IL-18 and tumor necrosis factor superfamily 14 have been shown to not be overexpressed in lipedema as highlighted in two of our previous studies, which included the same tissue samples investigated in the work presented here [7,27,44]. Of these cytokines, IL-11 is known to regulate adipogenesis and is mostly secreted by stromal vascular cells, which are present in increased numbers in the adipose tissue of lipedema patients [45,46]. Other cytokines involved in lipedema are VEGF-A, VEGF-C and VEGF-D, which affect angiogenesis and lymphangiogenesis [8,9]. Nevertheless, the search for a lipedema-specific biomarker is still ongoing. Another seemingly typical feature of lipedema is the low-grade inflammation characterized by the infiltration of M2-polarized macrophages [9], which is distinct from the M1 polarization in obesity [10]. We were able to highlight this in a previous study by detecting significantly increased gene expression levels of CD163 within the same lipedema tissue samples [9]. Building on these results and using the same cohort, we investigated the M2 macrophage levels using cytometry by time-of-flight and IHC and demonstrated significantly increased numbers of M2/CD163+ macrophages in lipedema compared with the control group [47].

Our present work illustrates an initial study to elucidate the role of the MIF superfamily in lipedema and to increase the understanding of lipedema. The current study is limited by a low number of enrolled patients and by exclusively selecting lipedema stages II and III, which does not present the whole spectrum of this disease. To compensate for these limitations, we chose matched characteristics such as BMI and age in both of the analyzed groups to create homogeneous and comparable populations [9,47]. Also, the study is of descriptive nature in its current form as it does not elaborate on the functional consequences of MIF family involvement in lipedema. Despite a great bulk of evidence that supports an active involvement of MIF-1 in adipose tissue inflammation and related diseases such as obesity, some studies were not able to substantiate this claim [11,48,49]. A context-dependent regulation of MIF-1 during adipose tissue inflammation may be likely as we showed a distinct upregulation of MIF-1 in inflamed adipose tissue from wounds earlier [14]. The precise effect of a potential MIF-1-CD74 link in lipedema necessitates further in-depth analysis. The binding of MIF-1 to CD74 in the presence of the coreceptor CD44 has several pro-inflammatory effects, such as the activation of MAPKs [50]. One prominent downstream mediator of the MIF-1-CD74 interaction is the downstream monocyte chemoattractant protein 1 (MCP-1) upregulation, e.g., through the p38 MAPK [50]. MCP-1 in return is a key chemokine secreted by macrophages that induces ASC proliferation and at the same time inhibits adipogenesis, which may play a role in lipedema [51]. Other downstream molecules or pathways that may play a role in lipedema include p-NF-κB and p-STAT3 as both have known correlations with obesity and adipose inflammation. In obesity, fatty acids are elevated, which in turn activate the expression of NF-κB-associated genes/cytokines such as IL-6 [52]. The serum level of lipids in lipedema are significantly increased, and IL-6 has been shown to be expressed in lipedema; however, it is not overexpressed when compared with obese patients [7,27], and it can activate p-STAT3 in the adipose tissue [53], which again can contribute to the production of pro-inflammatory cytokines and chemokines such as MCP-1 and IL-6, which can further perpetuate inflammation in adipose tissue. Therefore, a correlation between lipedema and these pathways/downstream molecules is very probable and could be of interest for future investigations, which was not possible in our study due to our very limited sample supply.

Lipedema seems to have a distinct systemic cytokine profile; however, to date, there is no specific biomarker for the diagnosis. The MIF superfamily is systemically measurable and has been described as a possible biomarker in sepsis and cancer. However, we do not believe that MIF-1 or CD74 will fill this role when it comes to lipedema because it is ubiquitously expressed in the presence of inflammation and because the specific inflammatory process seems to be localized in the adipose tissue of the affected limbs. Nevertheless, the serum levels of the MIF superfamily in lipedema should be investigated, and our results need to be supported by a larger sample size. Our discovery might, however, very well be important for the further understanding of lipedema.

## 5. Conclusions

In conclusion, we showed that MIF-1 mRNA, CD74 mRNA and their cellular levels are increased in the subcutaneous adipose tissue of lipedema patients, whereas MIF-2 expression remains unchanged. Furthermore, we demonstrated that CXCR2 expression was increased in IHC results, especially in the control group, which almost exclusively included obese patients. Staining of CXCR4 contrarily showed a decreased expression in both groups. These results may indicate an involvement of MIF-1 in the inflammatory process and recruitment of inflammatory cells and potentially also in the macrophage polarization in lipedema, possibly representing yet another piece in the puzzle for understanding the pathophysiology of lipedema. As the present study is purely descriptive, specific MIF-1 and CD74 action must be dissected in additional experiments.

## Figures and Tables

**Table 1:** Summary of patient data.

**Number of cases**	22	*p*-value
Lipedema group	11	
Control group	11	
**Sex**		
Female	22	
Male	0	
**Mean age (years)**		0.9824
Lipedema group	47.18, 95%CI: 41.20–53.35	
Control group	47.27, 95%CI: 40.43–53.93	
**Mean weight (kg)**		0.4603
Lipedema group	78.00, 95%CI: 70.26–95.30	
Control group	82.88, 95%CI: 70.96–85.04	
**Mean BMI (kg/m^2^)**		0.7530
Lipedema group	27.98, 95%CI: 25.12–32.21	
Control group	28.61, 95%CI: 26.15–30.05	
**Lipedema stage**		
Stage I	0	
Stage II	5	
Stage III	6	
Stage IV	0	

## Data Availability

The data presented in this study are available within the manuscript and the Supplementary Materials Table S1 and Figure S1.

## Supplementary Materials

The following supporting information can be downloaded at: https://www.mdpi.com/article/10.3390/metabo13101105/s1, Figure S1: The linear regression analysis was performed, and the Pearson correlation coefficient (r) and *p*-values (two-tailed) were determined to establish if there was a correlation between BMI and MIF-1 as well as CD74. There was no significant correlation between BMI and the RNA expression of MIF-1 and CD74. Table S1: Primers used for quantitative real time PCR.
