# Supplementary material for: Involvement of the Macrophage Migration Inhibitory Factor (MIF) in Lipedema
Source: Metabolites. 2023 Oct 23;13(10):1105. doi: 10.3390/metabo13101105 (PMC10608777; doi:10.3390/metabo13101105)
Supplement: Supplementary file 1 [file metabolites-13-01105-s001.zip › metabolites-2631049-supplementary.pdf]

Table S1. Primers used for quantitative real time PCR.

Gene	F / R	Primer sequence 5′ – 3′
	F	TGGTATCGTGGAAGGACTCATGAC
human *GAPDH*	R	ATGCCAGTGAGCTTCCCGTTCAGC
	F	TGTGCTCGCGCTACTCTCTCT
human *hß2M*	R	CGGATGGATGAAACCCAGACA
	F	AGGTGACTGTCAGTTTGTCC
human *CD74*	R	TTTCCATCCTGGTGACTCTG
	F	CCGGACAGGGTCTACATCAACTATTAC
human *MIF*	R	TAGGCGAAGGTGGAGTTGTTCC
	F	CGCCCACTTCTTTGAGTTTC
human *MIF-2*	R	GGAAGAAGCAGCCAGTTCAC

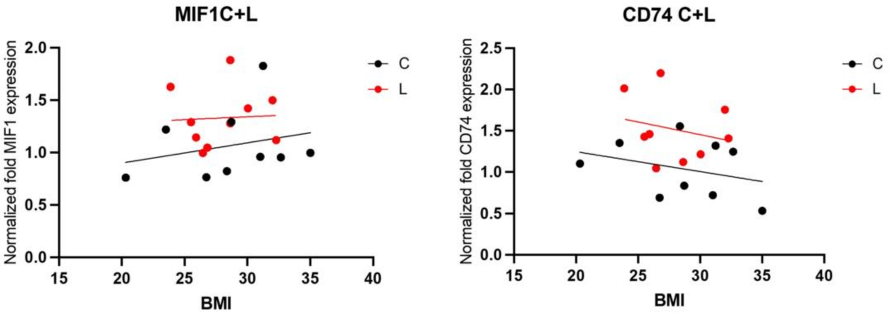

Figure S1: The linear regression analysis was performed, and the Pearson correlation coefficient (r) and p-values (two-tailed) were determined to establish if there was a correlation between BMI and MIF-1 as well as CD74. There was no significant correlation between BMI and the RNA expres-sion of MIF-1 and CD74.
